# 

**Published:** 1887-02

**Authors:** 


### CONTENTS, FEB. 1887, (VOL. II, NO. VIII.)

ORIGINAL ARTICLES—Obstetrics amongst the Eskimo: by Past-Assistant Surgeon H. M. Yeamans, M. H. S., Galveston, Texas .................................. 305

Osteo-Sarcoma below Trochanter Major: Resection in the Continuity; Cure, B. E. Hadra, M. D., Austin, Tex. 309

The Vålue of Electricity in Placenta Prævia. By W. M. Powell, M. D. Albany, Texas ........................ 312

Complicated Rupture of Urethra: By R. Menger, M. D., San Antonio, Texas .............................. 316

Veratrum in Puerperal Convulsions: By A. H. McCord, M. D., Rusk, Texas ................................ 31

Poisoning by Phytolacca: by J. N. Mendenhall, M. D., Plano, Texas .................................... 319

CORRESPONDENCE—That Criticism: (Dr. Briggs' Reply to Dr. Wallace) .................................... 329

EDITORIAL—The Coming Meeting of the Texas State Medical Association .................................. 332

Tall Oaks from Little Acorns ................................

Habitual Constipation ................................ 334

SOCIETY NOTES—Address of the President of the Texas State Medical Association to the Medical Profession 338

MEDICAL NEWS AND MISCELLANY—Bill to Create Local Board of Health; Cholera; Snowed Under; The Amende; The Pharmacy Bill; Killed, Dr. W. H. Thompson; Hardly Fair; Ship Island Quarantine; The Report on Texas Surgery; An unjust Imputation; Errata; Personal; A Brilliant Hit; The Prize Essay Again; The Changes at the Lunatic Asylum; Oh, Where shall Rest be found; He said it; Wanted; Hard Up; National Adulteration Bill; A Comedy of Errors; How Cholera was carried to Buenos Ayres; The new State Health Officer and other Appointees; Dr. McFarland, Dr. Wallace, Dr. Thompson, Drs. Preston and Davis; A Medical College in Texas ........................ 340–350

BIBLIOGRAPHY—Paralyses. Central, Bulbar and Spinal .... 350

A Treatise on the Practice of Medicine. Bartholow .... 351

Books and Phamplets received ........................ 351

Diseases of the Lungs and Pleura .................... 351

Diseases of the Blood and Nutrition .................. 352

The Best Antiseptic, Listerine ........................ 352

ADVERTISERS' NOTICES—Willians' Oxygen Apparatus. The J. P. Bush Manufacturing Co. C. N. Crittenden's Colden's Beef. Mellen's Food. Scott & Bowne. Bromidia. Hosfords Acid Phosphate in Skin Diseases. Over-Dosed Physicians ........................ 353–354

